# Systemic Inflammatory and Oxidative–Metabolic Alterations in Rosacea: A Cross-Sectional Case–Control Study

**DOI:** 10.3390/diagnostics16020246

**Published:** 2026-01-12

**Authors:** Mustafa Esen, Abdullah Demirbaş, Esin Diremsizoglu, Revşa Evin Canpolat Erkan

**Affiliations:** 1 Department of Dermatology, Faculty of Medicine, Dicle University, 21280 Diyarbakır, Türkiye; 2 Department of Dermatology, Faculty of Medicine, Kocaeli University, 41001 Kocaeli, Türkiye; 3 Department of Medical Biochemistry, Faculty of Medicine, Dicle University, 21280 Diyarbakır, Türkiye

**Keywords:** rosacea, systemic inflammation, oxidative stress, platelets, sirtuins, mitochondrial dysfunction, biomarkers, cardiometabolic risk

## Abstract

**Background/Objectives:** Rosacea increasingly appears to involve systemic immune and metabolic disturbances rather than isolated cutaneous inflammation. To evaluate inflammatory, platelet, and oxidative–metabolic biomarkers in rosacea and explore their interrelations. **Methods:** 90 patients with rosacea and 90 healthy controls were evaluated for hematologic inflammatory indices—neutrophil-to-lymphocyte ratio (NLR), platelet-to-lymphocyte ratio (PLR), systemic immune–inflammation index (SII), pan-immune–inflammation value (PIV), mean platelet volume (MPV), and C-reactive protein (CRP)—along with oxidative–metabolic regulators including sirtuin 1 (SIRT1), sirtuin 3 (SIRT3), visfatin, and irisin. Logistic regression and receiver operating characteristic (ROC) analyses were used to identify independent predictors of rosacea, while inter-marker associations were evaluated using Spearman’s rank correlation. **Results:** Rosacea patients showed higher NLR, PLR, SII, PIV, MPV, CRP, and LDL cholesterol (*p* < 0.05) and lower SIRT1, SIRT3, visfatin, and irisin (*p* < 0.01). MPV independently predicted rosacea (OR = 7.24; AUC = 0.827), whereas SIRT1 inversely correlated with disease risk. SIRT1, SIRT3, and visfatin showed inverse correlations with HbA1c and waist-to-height ratio, while fasting glucose and HOMA-IR remained within normal ranges. **Conclusions:** Rosacea exhibits dual systemic activation, an inflammatory–platelet and an oxidative–metabolic axis bridging immune dysregulation, mitochondrial stress, and vascular dysfunction. Recognition of these pathways highlights the potential of redox-targeted and metabolic interventions beyond symptomatic treatment.

## 1. Introduction

Rosacea is a chronic, relapsing inflammatory dermatosis characterized by centrofacial erythema, papules, and pustules arising from a multifactorial interplay of vascular, neuroimmune, and microbial mechanisms [1,2,3]. Aberrant activation of innate immunity—particularly through Toll-like receptor-2 (TLR-2) and cathelicidin (LL-37) pathways—triggers downstream cytokine cascades, Th1/Th17 polarization, and endothelial dysfunction [4,5]. Recent molecular evidence implicating the STING and NLRP3–IL-1β axes further links cutaneous immune dysregulation to systemic inflammation [6,7], suggesting that rosacea may represent a chronic inflammatory vasculopathy rather than a disease confined to the skin.

Hematology-derived indices such as the neutrophil-to-lymphocyte ratio (NLR), platelet-to-lymphocyte ratio (PLR), and systemic immune-inflammation index (SII) have emerged as accessible markers of systemic immune activation. Elevated values have been reported in rosacea and other inflammatory dermatoses, supporting a role for circulating leukocytes and platelets in disease pathophysiology [8,9,10]. Platelets, in particular, can amplify vascular inflammation by releasing serotonin, platelet factor-4, and pro-oxidant mediators [11,12], potentially linking cutaneous inflammation with microvascular reactivity. These peripheral blood indices serve as integrated markers of systemic immune activation. NLR and PLR reflect the balance between neutrophil-driven innate responses and lymphocyte-mediated regulation, while composite measures such as SII and PIV incorporate platelet activity, providing a broader representation of thrombo-inflammatory burden [8]. Their use in inflammatory dermatoses has been linked to endothelial activation, oxidative stress, and microvascular dysregulation, making them biologically relevant candidates for assessing potential systemic involvement in rosacea [9,10].

Beyond inflammation, accumulating epidemiologic evidence suggests that rosacea is associated with cardiometabolic alterations, including dyslipidemia, hypertension, and low-grade endothelial dysfunction [13]. These associations are thought to arise from shared mechanisms—such as TRPV-mediated neurovascular hyperreactivity, oxidative stress, and chronic systemic inflammation—that bridge cutaneous and metabolic pathways [14,15,16].

Oxidative imbalance further contributes to vascular and inflammatory dysregulation in rosacea. Sirtuins (SIRT1, SIRT3)—NAD^+^-dependent regulators of mitochondrial metabolism—suppress NF-κB–driven cytokine release and counteract oxidative stress [17]. Experimental activation of SIRT3 attenuates LL-37–induced oxidative injury in keratinocytes [18], underscoring its potential role in counteracting mitochondrial stress. However, circulating sirtuin dynamics in rosacea have yet to be systematically characterized. In addition, metabolic mediators such as visfatin and irisin, bridging cellular energy metabolism and immune signaling, are well documented in other chronic inflammatory dermatoses [19], but their relevance to rosacea pathophysiology has not been elucidated.

Despite increasing recognition of rosacea as a systemic inflammatory disorder, the interconnections between immune activation, oxidative stress, and metabolic dysfunction remain poorly defined. The present study therefore aimed to integrate hematologic inflammatory indices with oxidative–metabolic regulators (SIRT1, SIRT3, visfatin, and irisin) to determine whether rosacea represents a broader systemic inflammatory phenotype rather than a skin-limited disease. By evaluating these multidimensional biomarkers in parallel, we sought to elucidate mechanistic links bridging cutaneous inflammation, redox imbalance, and cardiometabolic risk.

## 2. Methods

### 2.1. Study Design and Participants

This cross sectional case–control study enrolled 90 adults with clinically confirmed rosacea and 90 age-matched healthy controls who attended the dermatology outpatient clinic of a tertiary hospital between March and October 2025. Diagnoses and clinical subtypes—erythematotelangiectatic (ETR) or papulopustular (PPR)—were established by board-certified dermatologists according to National Rosacea Society criteria [20]. Eligible participants were 18–65 years old and had received no topical or systemic therapy in the preceding two months. Exclusion criteria comprised chronic renal or hepatic disease, active infection, autoimmune or neoplastic disorders, endocrine disease (e.g., Cushing’s syndrome, polycystic ovary syndrome), diabetes mellitus, hypertension, pregnancy, and use of corticosteroids, retinoids, immunosuppressants, biologics, lipid-lowering agents, or systemic anti-inflammatory drugs within three months. Controls were healthy volunteers without any dermatologic, systemic, or metabolic disorders.

Demographic and anthropometric data (age, sex, height, weight, waist and hip circumference, blood pressure, smoking and alcohol status, and family history) were recorded. Body mass index (BMI) was calculated as weight/height^2^ (kg/m^2^), waist-to-hip ratio (WHR) as waist/hip, and waist-to-height ratio (WHtR) as waist/height. Rosacea severity was graded according to the National Rosacea Society clinical scoring system (2004) [20].

### 2.2. Laboratory Assessments

After at least 12 h of fasting, venous blood samples were obtained from the brachial vein into serum separator and EDTA tubes. Samples were centrifuged at 3500 rpm for 5 min, and serum aliquots were immediately analyzed for routine biochemistry (glucose, insulin, HbA1c, lipid profile, urea, creatinine, AST, ALT, CRP) using automated spectrophotometry. HbA1c was measured by high-performance liquid chromatography, and insulin by electrochemiluminescence. Estimated glomerular filtration rate (eGFR) was calculated using the 2021 CKD-EPI formula [21].

Complete blood counts were analyzed automatically, and derived indices were calculated as follows:

NLR = neutrophils/lymphocytes; PLR = platelets/lymphocytes; MLR = monocytes/lymphocytes; SII = (platelets × neutrophils)/lymphocytes; SIRI = (neutrophils × monocytes)/lymphocytes; PIV = (neutrophils × platelets × monocytes)/lymphocytes.

For oxidative–metabolic markers, 10 mL of serum was centrifuged at 1500× *g* for 20 min and stored at −80 °C until analysis. Serum SIRT1, SIRT3, irisin, and visfatin concentrations were measured using commercial enzyme-linked immunosorbent assay (ELISA) kits (Human Visfatin ELISA Kit, Cat. No. E0025Hu; Human Irisin ELISA Kit, Cat. No. E3253Hu; Human Sirtuin-1 ELISA Kit, Cat. No. E2557Hu; Human Sirtuin-3 ELISA Kit, Cat. No. E2559Hu; Bioassay Technology Laboratory, Zhejiang, China). All assays were performed according to the manufacturer’s instructions using a BioTek ELx50 microplate washer and a BioTek ELx800 microplate reader (BioTek Instruments, Inc., Winooski, VT, USA). Insulin resistance was estimated as HOMA-IR = [fasting insulin × glucose/405], and the triglyceride–glucose (TyG) index as ln (triglyceride × glucose/2). Metabolic syndrome was defined by NCEP-ATP III criteria [22].

### 2.3. Statistical Analysis

Normality was assessed using the Shapiro–Wilk test. Continuous variables were expressed as mean ± SD or median [IQR], and categorical variables as *n* (%). Group comparisons employed independent t-test or Mann–Whitney U test for continuous data and χ^2^ test for categorical data.

Multivariable logistic regression identified independent predictors of rosacea after adjusting for age, sex, BMI, smoking, alcohol and LDL. Waist-to-height ratio (WHtR) and total cholesterol were evaluated but not included in the multivariable models due to multicollinearity (VIF > 5). Each biomarker was entered separately to avoid multicollinearity. Adjusted odds ratios (OR) and 95% CIs were reported, with model fit evaluated by Nagelkerke R^2^ and Hosmer–Lemeshow test. Diagnostic accuracy was examined by receiver operating characteristic (ROC) analysis (AUC, Youden index, sensitivity, specificity).

Spearman’s correlations assessed relationships among inflammatory (NLR, PLR, SII), oxidative–metabolic (SIRT1, SIRT3, visfatin, irisin), and clinical variables (BMI, CRP, lipids). Correlations were classified as weak (ρ < 0.3), moderate (ρ 0.3–0.7), or strong (ρ > 0.7). To account for multiple testing within the predefined biomarker family (NLR, PLR, SII, PIV, MPV, CRP, SIRT1, SIRT3, visfatin, irisin) and correlation analyses, the Benjamini–Hochberg false discovery rate (FDR) correction was applied. Analyses were performed using SPSS v26 (IBM Corp., Armonk, NY, USA); *p* < 0.05 was considered statistically significant.

### 2.4. Ethical Considerations

The study received approval from the Institutional Review Board (protocol code 13; date of approval 18 December 2024). The research was conducted in accordance with the principles of the Declaration of Helsinki.

## 3. Results

### 3.1. Group Comparisons Between Rosacea and Controls

A total of 180 participants were analyzed (90 rosacea, 90 controls). Patients with rosacea had higher median age (42 vs. 27 years, *p* < 0.001), BMI, and waist-to-height ratio (*p* < 0.001 for both). Median disease duration was 24 months (IQR 12–84); 65.6% had erythematotelangiectatic (ETR) subtype and 52.2% had moderate severity. Alcohol use was less frequent in rosacea (*p* = 0.012), whereas smoking and metabolic-syndrome prevalence were comparable (both 30%).

**Figure 1:**
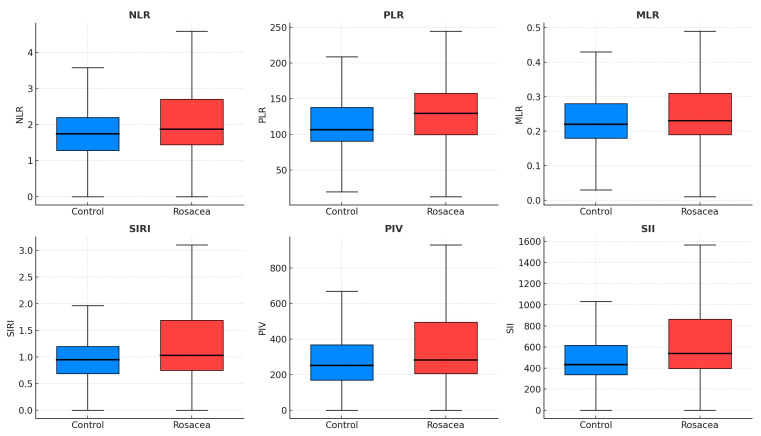
Boxplots showing inflammatory indices in rosacea and control groups. Median and interquartile ranges are displayed for NLR, PLR, MLR, SIRI, PIV, and SII. Elevated MPV, NLR, PLR, SII, and PIV indicate systemic immune–platelet activation in rosacea. Blue boxes = Controls; Red boxes = Rosacea. NLR, neutrophil-to-lymphocyte ratio; PLR, platelet-to-lymphocyte ratio; MLR, monocyte-to-lymphocyte ratio; SII, systemic immune-inflammation index; SIRI, systemic inflammation response index; PIV, pan-immune-inflammation value.

**Figure 2:**
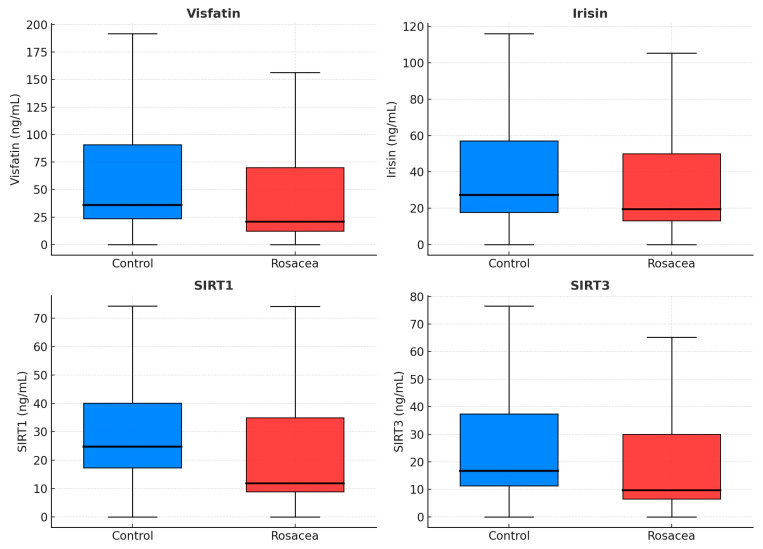
Boxplots of oxidative and energy-regulation markers in rosacea and controls. Reduced SIRT1, SIRT3, visfatin, and irisin levels suggest mitochondrial stress and impaired metabolic regulation. Blue boxes = Controls; Red boxes = Rosacea. SIRT1, sirtuin-1; SIRT3, sirtuin-3.

Total and LDL cholesterol were higher in rosacea (*p* < 0.001 each), while fasting glucose, insulin, HOMA-IR, TyG index, and metabolic-syndrome rates did not differ. CRP was elevated (2.41 vs. 1.29 mg/L, *p* < 0.001). Hematologic indices—NLR, PLR, SII, PIV, and MPV—were significantly increased (*p* = 0.012, 0.003, 0.005, 0.033, and <0.001) (Figure 1), whereas MLR and SIRI did not differ. Circulating visfatin, irisin, SIRT1, and SIRT3 were lower in rosacea (all *p* ≤ 0.011) (Table 1, Figure 2).

### 3.2. Independent Predictors of Disease

To assess whether these differences were independent of demographic and metabolic variables, logistic regression models were adjusted for age, sex, BMI, smoking, alcohol use, and LDL. Higher NLR (OR 1.59, 95% CI 1.10–2.30; *p* = 0.013), PLR (OR 1.009, 95% CI 1.002–1.016; *p* = 0.018), and SII (OR 1.001, 95% CI 1.000–1.002; *p* = 0.012) predicted rosacea independently. MPV showed the strongest effect (OR 7.09, 95% CI 3.66–13.74; *p* < 0.001) with model accuracy 82.2%, whereas PIV showed only borderline significance (*p* = 0.047).

Among oxidative–metabolic markers, only SIRT1 independently predicted rosacea (OR 1.014, 95% CI 1.003–1.026; *p* = 0.013); visfatin, irisin, and SIRT3 were not significant (*p* > 0.05). The MPV × SIRT1 interaction was nonsignificant (*p* = 0.335) (Table 2).

### 3.3. Diagnostic Performance of Inflammatory Markers

Receiver-operating characteristic analysis demonstrated that MPV showed the strongest independent association within this dataset (AUC 0.827, 95% CI 0.768–0.887; *p* < 0.001; sensitivity 85.6%, specificity 77.8%). NLR, PLR, and SII showed moderate accuracy (AUC 0.608–0.629, all *p* < 0.05) (Supplementary Figure S1).

SIRT1 exhibited an inverse pattern (AUC 0.387, *p* = 0.009), consistent with its lower serum levels in rosacea. Combining MPV with inflammatory ratios further improved overall discrimination (Table 3).

### 3.4. Associations Between Oxidative–Metabolic Biomarkers and Metabolic Parameters

Although the frequency of metabolic syndrome did not differ in the rosacea and control groups (27 [30.0%] vs. 27 [30.0%]; *p* = 1.000), correlation analysis revealed several significant associations between oxidative–metabolic biomarkers and metabolic variables. SIRT1, SIRT3, and visfatin correlated inversely with HbA1c (ρ = −0.18 to −0.19, *p* = 0.011–0.019) and waist-to-height ratio (ρ = −0.14 to −0.19, *p* = 0.016–0.069), whereas no associations were observed with HOMA-IR or TyG index (*p* > 0.30). Irisin showed no significant relationships (*p* > 0.05). However, none of these nominal correlations remained statistically significant after Benjamini–Hochberg FDR correction (Supplementary Table S1). After adjustment for age, sex, and BMI, none of these correlations remained significant (Supplementary Table S2).

### 3.5. Subtype Differences in Systemic Inflammatory, Oxidative, and Metabolic Markers

Comparison between erythematotelangiectatic (ETR) and papulopustular (PPR) rosacea subtypes revealed no significant differences in inflammatory or oxidative indices except for a higher HDL cholesterol level in the PPR group (median 63 vs. 53 mg/dL, *p* = 0.005 (Supplementary Table S3).

## 4. Discussion

This study suggests that rosacea may be associated with a distinct systemic profile involving inflammatory, oxidative, and metabolic alterations. Compared with healthy controls, patients exhibited significantly elevated NLR, PLR, SII, PIV, MPV, CRP, and lipid levels, together indicating low-grade systemic inflammation extending beyond cutaneous involvement. These findings align with previous hematologic studies [8,10,23]. Elevated SII and PIV may reflect concurrent activation of neutrophils, lymphocytes, and platelets, mechanisms that have been proposed to contribute to endothelial dysfunction and vascular reactivity in rosacea. Collectively, these data reinforce the concept of a platelet–neutrophil inflammatory axis linking cutaneous inflammation with systemic vascular reactivity. Mechanistically, LL-37-driven TLR2 signaling and neutrophil infiltration may contribute to Th1/Th17 polarization and cytokine release [4,5,6], whereas platelet-derived serotonin, histamine, and platelet factor-4 further amplify vasodilation and endothelial dysfunction [11,12].

In the present study, classical inflammatory ratios (NLR, PLR) and composite indices (SII, PIV) were consistently elevated in rosacea, a pattern broadly compatible with current models of neuroimmune–vascular dysregulation. Elevations in NLR and PLR may represent a shift toward neutrophil-predominant innate immune activity with relative lymphocyte suppression, an immune profile described in prior clinical studies of rosacea [8,9,10]. Composite indices such as SII and PIV, which integrate neutrophils, lymphocytes, and platelets, may provide a broader reflection of thrombo-inflammatory activity. Platelet–neutrophil interactions have been proposed to contribute to endothelial dysfunction and vascular reactivity in rosacea [12,14]. The parallel elevation of these markers in this cohort is therefore compatible with the hypothesis that rosacea may involve systemic inflammatory or thrombo-inflammatory activity beyond cutaneous involvement.

Among inflammatory indices, MPV showed the strongest independent association with rosacea and demonstrated exploratory discriminative ability within this dataset (AUC = 0.827). Larger platelets are metabolically and pro-inflammatory more active, releasing higher levels of cytokines and chemokines [24], which may contribute to vascular and inflammatory activity. Elevated MPV has been consistently reported in rosacea and other chronic inflammatory dermatoses [8,9,10], and is also recognized as a marker of cardiovascular and metabolic morbidity [25], raising the possibility of shared systemic pathways. In this cohort, MPV also showed weak inverse correlations with CRP and SII, which may reflect increased platelet consumption during periods of inflammation, as suggested in other inflammatory states [24]. Taken together, these observations support the hypothesis that MPV may reflect fluctuations in systemic inflammatory load, although external validation is required and all ROC estimates should be interpreted as exploratory.

Beyond inflammation, lower circulating SIRT1, SIRT3, visfatin, and irisin levels may be compatible with disturbances in mitochondrial or redox pathways, although these associations weakened after adjustment and should be interpreted cautiously. Sirtuins are NAD^+^-dependent deacetylases that maintain mitochondrial biogenesis, redox balance, and NF-κB signaling [17,18]. Experimental SIRT3 activation has been shown to protect keratinocytes from LL-37-induced oxidative injury [18], while sirtuin deficiency promotes mitochondrial ROS accumulation and cytokine release. Reduced sirtuin, visfatin, and irisin levels have also been observed in metabolic and cardiovascular disorders [19], supporting their role as early indicators of oxidative–metabolic imbalance. Our findings therefore extend previous evidence of mitochondrial dysfunction and redox imbalance to rosacea, suggesting a systemic oxidative–metabolic component underlying cutaneous inflammation.

The concomitant elevation of LDL and total cholesterol, with preserved glycemic indices, supports the concept that rosacea resides within the cardiometabolic continuum. Prior meta-analyses report increased risks of hypertension and dyslipidemia but inconsistent associations with diabetes or ischemic heart disease [13]. This profile suggests subclinical vascular dysregulation rather than overt metabolic pathology. Shared mechanisms such as NLRP3–IL-1β activation, lipid oxidation, and endothelial dysfunction likely contribute to both rosacea and cardiometabolic disease [6,16]. Dysregulated lipoprotein handling contributes to atherogenesis, consistent with the elevated LDL and CRP observed here. Statins exert pleiotropic anti-inflammatory effects through inhibition of monocyte and Th1 activation [26]; whether these benefits extend to rosacea warrants further study. Hypertension also appears to share neurovascular roots with rosacea, with TRPV-mediated vascular hyperreactivity and sympathetic signaling implicated in both conditions [14,15]. Certain antihypertensive agents exert opposite effects on rosacea vasoreactivity. Dihydropyridine calcium-channel blockers have been reported to exacerbate facial flushing and erythema [27], whereas non-selective β-blockers—particularly carvedilol—can significantly reduce vasomotor symptoms and persistent erythema, as supported by recent clinical observations [28,29]. These pharmacologic observations reinforce the concept of a shared neurovascular substrate rather than coincidental comorbidity.

Although several oxidative–metabolic markers (SIRT1, SIRT3, visfatin) showed nominal inverse correlations with HbA1c and waist-to-height ratio, these associations did not remain statistically significant after FDR adjustment and were attenuated further after controlling for age, sex, and BMI. Therefore, these relationships should be interpreted as exploratory and hypothesis-generating rather than indicative of a definitive metabolic pattern. Sirtuins (SIRT1 and SIRT3) are NAD^+^-dependent deacetylases that regulate mitochondrial redox balance, endothelial nitric-oxide signaling, and insulin sensitivity [17,18], while visfatin and irisin act as adipomyokines modulating glucose utilization and energy homeostasis [19]. Depletion of these regulators may impair mitochondrial ROS detoxification, activate stress kinases, and inhibit Akt phosphorylation, thereby attenuating insulin signaling and promoting endothelial dysfunction [30]. After adjustment for age, sex, and BMI, these associations lost significance, suggesting that reduced sirtuin and visfatin levels may reflect metabolic load–related oxidative stress rather than adiposity-independent mechanisms. These exploratory patterns may suggest a potential but unconfirmed oxidative–metabolic susceptibility in rosacea. Stronger evidence from longitudinal and mechanistic studies is required to determine whether such alterations have clinical or vascular relevance. Findings in the literature remain partly conflicting; prior research has proposed that sirtuins, visfatin, and irisin could serve as biomarkers of metabolic and cardiovascular risk [31,32,33]. In this context, the present results should be interpreted as hypothesis-generating rather than definitive, and they highlight the need for future work to clarify whether oxidative–metabolic changes contribute to the broader systemic profile observed in rosacea

No significant differences in inflammatory or oxidative markers were observed between erythematotelangiectatic and papulopustular subtypes, except for higher HDL cholesterol in the latter. This suggests that systemic inflammatory and oxidative profiles are largely shared across rosacea phenotypes.

This study has several limitations. Although multivariable logistic regression identified variables independently associated with rosacea, residual confounding cannot be fully excluded due to the observational cross-sectional design. Unmeasured factors including diet, stress, hormonal status, or medication use may still have influenced the observed associations. The sample size, while adequate for moderate effect detection, limited subgroup analyses by clinical subtype or disease duration. Moreover, reliance on peripheral blood biomarkers does not capture tissue-level or transcriptomic changes underlying inflammation and oxidative stress. Because this study is cross-sectional, temporal relationships and causal direction cannot be inferred. The findings should be viewed as associative and hypothesis-generating. Finally, the absence of direct endothelial or vascular imaging measures restricts mechanistic interpretation of the cardiometabolic findings.

## 5. Conclusions

The present findings support a multidimensional model of rosacea pathogenesis involving two interconnected systemic axes:

(1) an inflammatory–platelet axis (elevated NLR, PLR, SII, MPV, CRP) indicating immune activation and vascular dysfunction, and
(2) an oxidative–metabolic axis (reduced SIRT1, SIRT3, visfatin, irisin) reflecting mitochondrial stress and impaired energy regulation.

These pathways may help to conceptualize the coexistence of cutaneous inflammation and systemic features; however, the interpretations are exploratory and require confirmation in larger, prospectively designed studies. Easily accessible blood biomarkers such as MPV, NLR, may aid systemic risk assessment. Targeting redox balance through NAD^+^ precursors, sirtuin activators, or antioxidants could complement current therapies. Future studies should determine whether modulating systemic inflammation and oxidative stress can improve disease severity and long-term vascular outcomes.

## Figures and Tables

**Table 1:** Comparison of demographic, metabolic, and inflammatory parameters between rosacea and control groups.

Variable	Control (*n* = 90)	Rosacea (*n* = 90)	*p*-Value
**Age (years)**	27 [23–40]	42 [31–50]	**<0.001 ^b^**
**Sex (female)**	81 (90.0%)	83 (92.2%)	0.600 ^c^
**Height (cm)**	170.5 [163–177]	160 [158–165]	**<0.001 ^b^**
**Weight (kg)**	75 [59.8–81]	69 [64–80]	0.597 ^b^
**BMI (kg/m^2^)**	25.0 [21.5–27.8]	26.8 [24.7–29.4]	**<0.001 ^b^**
**Waist (cm)**	90 [78–100]	93.5 [85–102]	0.081 ^b^
**Hip (cm)**	102.6 ± 9.9	108.3 ± 10.8	**<0.001 ^a^**
**WHR**	0.84 ± 0.07	0.86 ± 0.09	0.205 ^a^
**WHtR**	0.50 ± 0.05	0.57 ± 0.08	**<0.001 ^a^**
**Systolic BP (mmHg)**	113 [105–125]	120 [110–129]	**0.014 ^b^**
**Diastolic BP (mmHg)**	75 [70–80]	80 [70–85]	**0.004 ^b^**
**Fitzpatrick type II–V**	II 8/III 33/IV 39/V 10	II 12/III 39/IV 33/V 6	0.423 ^c^
**Insulin (µIU/mL)**	13.3 [7.0–25.2]	9.9 [7.2–16.1]	0.162 ^b^
**Fasting Blood Glucose** **(mg/dL)**	90 [82–100]	93 [86–101]	0.227 ^b^
**HOMA-IR**	2.69 [1.44–6.79]	2.30 [1.50–3.90]	0.331 ^b^
**C-peptide(ng/mL)**	3.83 [2.44–7.05]	3.28 [2.38–4.94]	0.222 ^b^
**HbA1c (%)**	5.35 [5.10–5.60]	5.50 [5.30–5.80]	**0.005 ^b^**
**CRP (mg/L)**	1.29 [0.64–2.71]	2.41 [1.20–5.70]	<0.001 *^b^
**Urea (mg/dL)**	27 [23–33]	26 [22–30]	0.314 ^b^
**Kreatinin (mg/dL)**	0.82 [0.70–0.92]	0.64 [0.56–0.76]	**<0.001 ^b^**
**eGFR (CKD-EPI, mL/min/1.73 m^2^)**	106 [98–114]	114 [101–121]	**<0.001 ^b^**
**ALT (U/L)**	17 [12–22]	17.5 [13–24]	0.390 ^b^
**AST (U/L)**	21 [18–25]	20 [18–25]	0.863 ^b^
**Metabolic syndrome (NCEP-ATP III)**	27 (30.0%)	27 (30.0%)	1.000 ^c^
**Smoking**	33 (36.7%)	23 (25.6%)	0.107 ^c^
**Alcohol use**	12 (13.3%)	2 (2.2%)	**0.012 ^c^**
**Total cholesterol (mg/dL)**	177 [156–207]	194 [176–223]	**<0.001 ^b^**
**HDL (mg/dL)**	50.5 [42–62]	55 [47–64]	0.094 ^b^
**LDL (mg/dL)**	96.9 [78.4–119.8]	119.4 [98.8–137.8]	**<0.001 ^b^**
**VLDL (mg/dL)**	24.1 [17.4–33.4]	22.4 [15.8–36.2]	0.484 ^b^
**Triglycerides (mg/dL)**	139 [87–167]	113.5 [79–181]	0.753 ^b^
**TyG index**	8.55 [8.29–9.05]	8.58 [8.14–9.04]	0.639 ^b^
**AIP**	0.02 ± 0.29	−0.05 ± 0.26	0.363 ^a^
**WBC (×10^9^/L)**	7.33 [6.27–8.56]	7.68 [6.34–9.22]	0.334 ^b^
**Neutrophil (×10^9^/L)**	4.10 [3.11–5.02]	4.57 [3.30–5.75]	0.056 ^b^
**Lymphocyte (×10^9^/L)**	2.43 [1.95–2.99]	2.33 [1.82–2.68]	0.093 ^b^
**Platelet (×10^3^/µL)**	265.5 [232–327]	293.5 [251–337]	0.059 ^b^
**Monocyte (×10^9^/L)**	0.54 [0.48–0.65]	0.56 [0.47–0.64]	0.920 ^b^
**MPV (fL)**	8.91 [8.33–9.81]	10.20 [9.60–10.70]	<0.001 *^b^
**NLR**	1.74 [1.28–2.20]	1.87 [1.44–2.70]	0.012 *^b^
**PLR**	106.5 [90.5–137.8]	129.4 [99.6–157.6]	0.003 *^b^
**MLR**	0.22 [0.18–0.28]	0.23 [0.19–0.31]	0.132 ^b^
**SIRI**	0.95 [0.69–1.20]	1.03 [0.75–1.69]	0.069 ^b^
**PIV**	252.3 [168.9–368.9]	282.9 [205.9–495.3]	0.033 *^b^
**SII**	432.2 [338.6–615.9]	537.8 [396.1–864.5]	0.005 *^b^
**Visfatin (ng/mL)**	35.9 [23.7–90.9]	20.8 [12.3–77.3]	<0.001 *^b^
**Irisin (ng/mL)**	27.3 [17.8–57.1]	19.4 [13.1–67.0]	0.011 *^b^
**SIRT1 (ng/mL)**	24.8 [17.3–40.1]	11.9 [8.9–71.7]	0.009 *^b^
**SIRT3 (ng/mL)**	16.7 [11.3–37.4]	9.7 [6.5–45.2]	0.001 *^b^
Family history **	—	Yes 4 (4.4%)/No 86 (95.6%)	—
Disease severity **	—	Mild 28 (31.1%)/Moderate 47 (52.2%)/Severe 15 (16.7%)	—
Disease duration (months) **	—	24 (12–84)	—
Clinical subtype **	—	ETR 59 (65.6%)/PPR 31 (34.4%)	—

* Significance determined after Benjamini–Hochberg false discovery rate (FDR) correction. All markers remained significant after FDR adjustment. ** Variables available only within the rosacea group. Values are expressed as mean ± SD for normally distributed, median [IQR] for non-normally distributed, and n (%) for categorical variables. ^a^ Independent samples *t*-test. ^b^ Mann–Whitney U test. ^c^ Chi-square test. BMI, body mass index; WHR, waist-to-hip ratio; WHtR, waist-to-height ratio; BP, blood pressure; HOMA-IR, homeostatic model assessment of insulin resistance; CRP, C-reactive protein; eGFR, estimated glomerular filtration rate; CKD-EPI, Chronic Kidney Disease Epidemiology Collaboration; ALT, alanine aminotransferase; AST, aspartate aminotransferase; AIP, atherogenic index of plasma; WBC, white blood cell count; NLR, neutrophil-to-lymphocyte ratio; PLR, platelet-to-lymphocyte ratio; MLR, monocyte-to-lymphocyte ratio; SII, systemic immune-inflammation index; SIRI, systemic inflammation response index; PIV, pan-immune-inflammation value; TyG, triglyceride–glucose index; LDL, low-density lipoprotein; HDL, high-density lipoprotein; VLDL, very-low-density lipoprotein; SIRT1, sirtuin-1; SIRT3, sirtuin-3; ETR, erythematotelangiectatic rosacea; PPR, papulopustular rosacea. Significant results (*p* < 0.05) are shown in bold.

**Table 2:** Multivariable logistic regression models for rosacea risk.

Variable	Adjusted OR (95% CI)	*p*-Value	Nagelkerke R^2^	Accuracy (%)
**Base model**			0.326	72.2
Age (years)	1.069 (1.032–1.106)	**<0.001**		
Sex (female)	1.48 (0.38–5.81)	0.571		
BMI (kg/m^2^)	1.099 (1.010–1.197)	**0.029**		
Smoking (yes)	0.56 (0.26–1.22)	0.143		
Alcohol (yes)	0.19 (0.04–1.02)	0.052		
LDL	1.008 (0.998–1.019)	0.121		
**Systemic inflammation markers**				
+NLR	1.594 (1.103–2.304)	**0.013**	0.376	75.6
+PLR	1.009 (1.002–1.016)	**0.018**	0.361	75.6
+SII	1.001 (1.000–1.002)	**0.012**	0.380	77.2
+MPV	7.09 (3.66–13.74)	**<0.001**	0.633	82.2
+PIV	1.001 (1.000–1.002)	0.047	0.352	73.9
**Oxidative/energy-regulation markers**				
+Visfatin	0.999 (0.995–1.004)	0.792	0.326	73.3
+Irisin	0.999 (0.991–1.007)	0.833	0.326	72.2
+SIRT1	1.014 (1.003–1.026)	**0.013**	0.366	72.8
+SIRT3	1.006 (0.994–1.018)	0.331	0.331	72.8
**Interaction model**				
+MPV × SIRT1	0.993 (0.980–1.007)	0.335	0.333	71.7

Base model adjusted for age, sex, BMI, smoking, and alcohol use. OR, odds ratio; CI, confidence interval; BMI, body mass index; NLR, neutrophil-to-lymphocyte ratio; PLR, platelet-to-lymphocyte ratio; SII, systemic immune-inflammation index; MPV, mean platelet volume; PIV, pan-immune-inflammation value; SIRT1, sirtuin-1; SIRT3, sirtuin-3. Significant results (*p* < 0.05) are shown in bold.

**Table 3:** ROC analysis of hematologic and inflammatory markers for discriminating rosacea from controls.

Marker	AUC (95% CI)	*p*-Value	Cut-Off (Youden)	Sensitivity (%)	Specificity (%)
**NLR**	0.608 (0.526–0.690)	0.012	≥1.54	56.7	61.1
**PLR**	0.629 (0.548–0.711)	0.003	≥105	63.3	58.9
**SII**	0.621 (0.540–0.702)	0.005	≥432	60.0	60.0
**MPV**	0.827 (0.768–0.887)	**<0.001**	≥8.9 fL	85.6	77.8
**SIRT1**	0.387 (0.295–0.480)	0.009	≤10.0	55.6	61.1

AUC, area under the curve; CI, confidence interval; NLR, neutrophil-to-lymphocyte ratio; PLR, platelet-to-lymphocyte ratio; SII, systemic immune-inflammation index; MPV, mean platelet volume; SIRT1, sirtuin-1. Cut-off values were determined using the Youden index. Significant results (*p* < 0.05) are shown in bold.

## Data Availability

The data supporting the findings of this study are available from the corresponding author upon reasonable request.

## Supplementary Materials

The following supporting information can be downloaded at: https://www.mdpi.com/article/10.3390/diagnostics16020246/s1, Figure S1: Receiver operating characteristic (ROC) curve for MPV in discriminating rosacea from controls; Table S1: Spearman’s correlations between oxidative–metabolic regulators and metabolic indices; Table S2: Unadjusted and age-, sex-, and BMI-adjusted correlations between oxidative–metabolic biomarkers and metabolic indices in rosacea patients. Table S3. Comparison of systemic inflammatory, oxidative, and metabolic markers between erythematotelangiectatic (ETR) and papulopustular (PPR) rosacea subtypes.
