# Supplementary material for: Systemic Inflammatory and Oxidative–Metabolic Alterations in Rosacea: A Cross-Sectional Case–Control Study
Source: Diagnostics (Basel). 2026 Jan 12;16(2):246. doi: 10.3390/diagnostics16020246 (PMC12839567; doi:10.3390/diagnostics16020246)
Supplement: Supplementary file 1 [file diagnostics-16-00246-s001.zip › Supplementary Figure.pdf]

Supplementary Figure S1. Receiver operating characteristic (ROC) curve for MPV in discriminating rosacea from controls. The area under the curve (AUC = 0.827, 95% CI 0.768–0.887; p < 0.001) indicates strong diagnostic accuracy, with 85.6% sensitivity and 77.8% specificity.

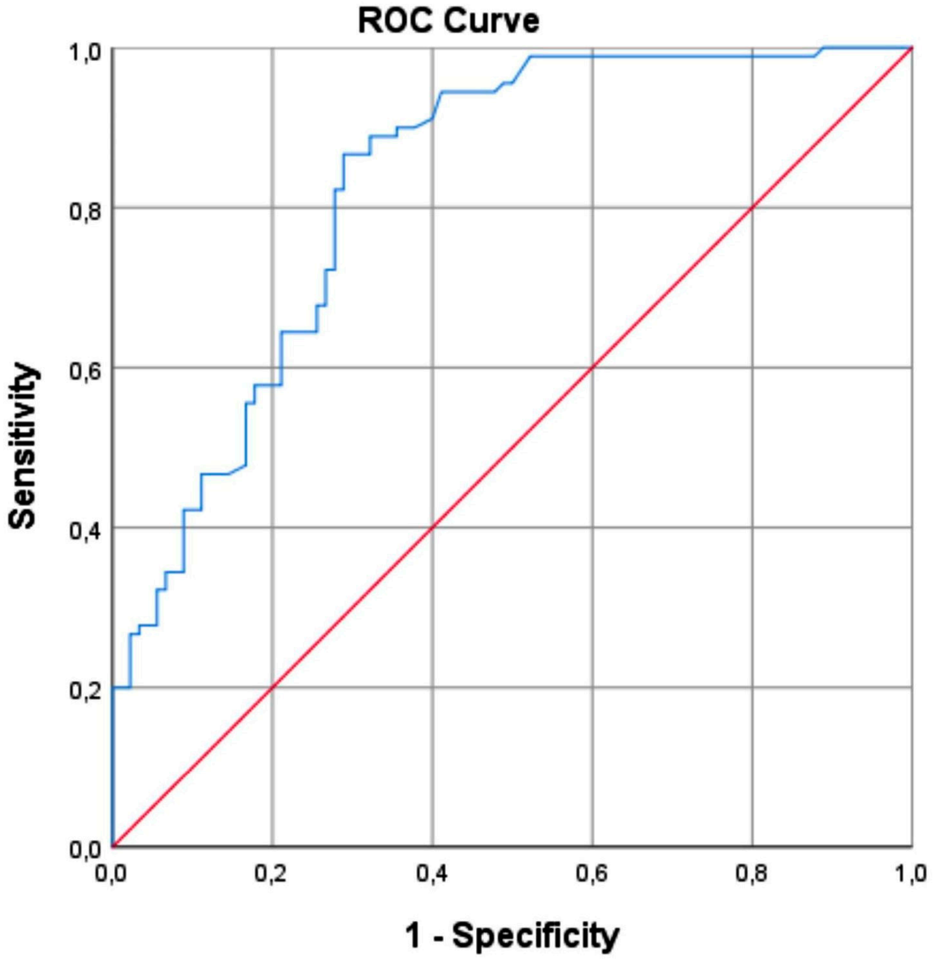

Area	Std. Error[a]	Asymptotic Sig.[b]	Asymptotic 95% Confidence Interval	
			Lower Bound	Upper Bound
,827	,031	,000	,768	,887
