# Supplementary material for: Systemic Inflammatory and Oxidative–Metabolic Alterations in Rosacea: A Cross-Sectional Case–Control Study
Source: Diagnostics (Basel). 2026 Jan 12;16(2):246. doi: 10.3390/diagnostics16020246 (PMC12839567; doi:10.3390/diagnostics16020246)
Supplement: Supplementary file 1 [file diagnostics-16-00246-s001.zip › Supplementary Tables.pdf]

Supplementary Table S1. Spearman's correlations between oxidative–metabolic regulators and metabolic indices

Biomarker	HOMA-IR	TyG index	HbA1c	LDL	Total chol	WHtR
**SIRT1**	–0.070 (p = 0.390; q = 0.660)	0.000 (p = 0.980; q = 0.990)	–0.182 (p = 0.014; q = 0.091)	–0.070 (p = 0.350; q = 0.646)	–0.050 (p = 0.510; q = 0.693)	–0.136 (p = 0.069; q = 0.276)
**SIRT3**	–0.020 (p = 0.810; q = 0.926)	0.080 (p = 0.290; q = 0.580)	–0.190 (p = 0.011; q = 0.091)	–0.110 (p = 0.160; q = 0.384)	–0.040 (p = 0.560; q = 0.707)	–0.179 (p = 0.016; q = 0.091)
**Visfatin**	0.000 (p = 0.990; q = 0.990)	0.100 (p = 0.190; q = 0.415)	–0.175 (p = 0.019; q = 0.091)	–0.120 (p = 0.120; q = 0.320)	–0.060 (p = 0.440; q = 0.660)	–0.189 (p = 0.011; q = 0.091)
**Irisin**	–0.050 (p = 0.520; q = 0.693)	0.030 (p = 0.740; q = 0.888)	–0.130 (p = 0.083; q = 0.285)	–0.060 (p = 0.430; q = 0.660)	0.010 (p = 0.910; q = 0.990)	–0.124 (p = 0.098; q = 0.294)

Spearman's rank correlation coefficients (ρ) are shown; p values in parentheses. Benjamini–Hochberg false discovery rate (FDR)–adjusted q-values were calculated for correlations. Several correlations were nominally significant; however, none remained significant after FDR correction (all $q \geq 0.091$). Thus, these associations should be interpreted as exploratory.

SIRT1, sirtuin 1; SIRT3, sirtuin 3; HbA1c, glycated hemoglobin; HOMA-IR, homeostasis model assessment of insulin resistance; TyG, triglyceride–glucose index; LDL, low-density lipoprotein cholesterol; Total chol, total cholesterol; WHtR, waist-to-height ratio.

Supplementary Table S2. Unadjusted and age-, sex-, and BMI-adjusted correlations between oxidative–metabolic biomarkers and metabolic indices in rosacea patients

Biomarker	Metabolic Parameter	ρ (Unadjusted)	p-value (Unadjusted)	ρ (Adjusted)*	p-value (Adjusted)*
**SIRT1**	HbA1c (%)	–0.182	0.014	–0.091	0.402
	WHtR	–0.136	0.069	+0.172	0.112
**SIRT3**	HbA1c (%)	–0.190	0.011	–0.082	0.451
	WHtR	–0.179	0.016	+0.179	0.097
**Visfatin**	HbA1c (%)	–0.175	0.019	–0.067	0.535
	WHtR	–0.189	0.011	+0.131	0.228

Biomarker	Metabolic Parameter	ρ (Unadjusted)	p-value (Unadjusted)	ρ (Adjusted)*	p-value (Adjusted)*
**Irisin**	HbA1c (%)	–0.130	0.083	—	—
	WHtR	–0.124	0.098	—	—

Partial (Spearman) correlations adjusted for age, sex, and body mass index (BMI).

ρ (rho) denotes Spearman's rank correlation coefficient.

HbA1c, glycated hemoglobin; WHtR, waist-to-height ratio; BMI, body mass index; SIRT1, sirtuin-1; SIRT3, sirtuin-3; Visfatin, nicotinamide phosphoribosyltransferase; Irisin, fibronectin type III domain-containing protein 5.

Supplementary Table S3. Comparison of systemic inflammatory, oxidative, and metabolic markers between erythematotelangiectatic (ETR) and papulopustular (PPR) rosacea subtypes

Marker	ETR median [IQR]	PPR median [IQR]	p-value
**Inflammatory indices**			
MPV (fL)	10.1 [9.6–10.5]	10.4 [9.5–10.9]	0.322
NLR	1.85 [1.38–2.57]	2.11 [1.47–2.91]	0.527
PLR	129.3 [95.5–157.6]	131.0 [114.0–165.1]	0.352
SII	518 [397–860]	577 [378–878]	0.463
CRP (mg/L)	3.32 [1.20–5.73]	2.30 [1.18–5.46]	0.829
MLR	0.22 [0.19–0.31]	0.26 [0.20–0.36]	0.348
SIRI	1.01 [0.72–1.63]	1.09 [0.85–2.00]	0.521
PIV	276.7 [207.9–472.2]	332.5 [199.8–675.2]	0.625
**Oxidative / energy-regulation markers**			
Visfatin (ng/mL)	20.8 [12.9–77.3]	17.3 [10.6–86.7]	0.335
Irisin (ng/mL)	19.1 [12.8–56.1]	23.5 [13.1–70.8]	0.895
SIRT1 (ng/mL)	11.9 [9.2–67.5]	13.5 [8.3–80.2]	0.976
SIRT3 (ng/mL)	9.0 [6.4–45.8]	11.1 [6.5–44.9]	0.760
**Metabolic and vascular parameters**			
BMI (kg/m²)	26.9 [25.3–29.7]	26.2 [24.2–29.4]	0.149
WHR	0.85 [0.81–0.93]	0.87 [0.79–0.93]	0.659
WHtR	0.57 [0.53–0.62]	0.58 [0.51–0.64]	0.875
Systolic BP (mmHg)	112 [102–120]	115 [110–125]	0.820

Marker	ETR median [IQR]	PPR median [IQR]	p-value
Diastolic BP (mmHg)	75 [70–80]	75 [70–80]	0.829
Total cholesterol (mg/dL)	191 [176–220]	202 [177–241]	0.162
LDL (mg/dL)	120 [101–131]	118 [98–152]	0.544
HDL (mg/dL)	53 [47–58]	63 [51–70]	**0.005**
Triglycerides (mg/dL)	118.7 [79–181]	107.0 [75–194]	0.505
VLDL (mg/dL)	23.6 [15.8–36.2]	21.4 [15.0–38.8]	0.607
AIP (log TG/HDL)	–0.03 [–0.18–0.21]	–0.13 [–0.29–0.16]	0.445
TyG index	8.63 [8.22–9.06]	8.47 [8.08–8.96]	0.445
Insulin (µIU/mL)	9.88 [6.37–17.79]	10.24 [7.70–13.40]	0.410
Fasting glucose (mg/dL)	93 [86–101]	91 [86–103]	0.922
HOMA-IR	2.17 [1.40–4.60]	2.35 [1.51–3.47]	0.543
C-peptide (ng/mL)	3.30 [2.38–5.27]	3.24 [2.35–4.40]	0.929
HbA1c (%)	5.60 [5.30–5.80]	5.40 [5.20–5.90]	0.680

Mann–Whitney U test (two-tailed) used for all comparisons. Bold indicates statistical significance ($p < 0.05$). MPV, mean platelet volume; NLR, neutrophil-to-lymphocyte ratio; PLR, platelet-to-lymphocyte ratio; SII, systemic immune-inflammation index; CRP, C-reactive protein; MLR, monocyte-to-lymphocyte ratio; SIRI, systemic inflammation response index; PIV, pan-immune-inflammation value; Visfatin, nicotinamide phosphoribosyltransferase; Irisin, fibronectin type III domain-containing protein 5; SIRT1, sirtuin-1; SIRT3, sirtuin-3; BMI, body mass index; WHR, waist-to-hip ratio; WHtR, waist-to-height ratio; BP, blood pressure; LDL, low-density lipoprotein; HDL, high-density lipoprotein; VLDL, very-low-density lipoprotein; AIP, atherogenic index of plasma; TyG, triglyceride–glucose index; HOMA-IR, homeostatic model assessment of insulin resistance; HbA1c, glycated hemoglobin.
